# The Influence of Different Drying Methods on Constituents and Antioxidant Activity of Saffron from China

**DOI:** 10.1155/2015/953164

**Published:** 2015-03-30

**Authors:** Yingpeng Tong, Xingyi Zhu, Yongqiu Yan, Ruoxi Liu, Feng Gong, Ling Zhang, Jiangning Hu, Ling Fang, Ruwei Wang, Ping Wang

**Affiliations:** ^1^College of Pharmaceutical Sciences, Zhejiang University of Technology, Collaborative Innovation Center of Yangtze River Delta Region Green Pharmaceuticals, Hangzhou 310014, China; ^2^Department of Biochemistry and Molecular Biology, University of Miami, Miller School of Medicine, Miami, FL 33124, USA; ^3^Zhejiang CONBA Pharmaceutical Co., Ltd., Hangzhou 310000, China

## Abstract

More and more saffron has been cultivated in China because of the increasing saffron demand, but no paper has studied the influence of drying methods on the quality of Chinese saffron. In this paper, three different dehydration treatments applied in actual production were evaluated: dehydration with electric oven, vacuum oven, and microwave. We determined that the highest quality of saffron will be obtained when fresh saffron is treated at higher temperatures (no more than 70°C) for a long time by electric oven drying and vacuum oven drying. In microwave drying, treatments at lower microwave power and longer time benefit the quality of saffron. In addition, the influence of the drying method on antioxidants in saffron is discussed. The correlation between individual saffron profiles and the antioxidant value was estimated by spectrum-effect relationships analysis.

## 1. Introduction

Saffron, the dried and dark-red stigmas of* Crocus sativus* L., is one of the most important spices in the world and has been mainly cultivated in Iran, Greece, Morocco, India, Spain, and Italy. Saffron is used mainly as a spice for flavoring and coloring food, and numerous studies have also shown saffron to be capable of having a variety of pharmacological effects, such as antihypertensive, anticonvulsant, antitussive, antigenotoxic, and cytotoxic effects and anxiolytic and aphrodisiac, antioxidant, antidepressant, anti-inflammatory, and relaxant activities. Saffron also improves memory and learning skills and increases blood flow in the retina and choroid. The antioxidant activity has been hypothesized to be one of the important mechanisms for the various pharmacological effects of saffron [1]. Saffron extracts and several compounds from saffron including crocins, crocetin, carotene, and safranal have exhibited different antioxidative activities both in vivo and in vitro [2].

The main active compounds in saffron are crocins, a group of glycoside derivatives from the carotenoid crocetin; terpenic aldehydes known as safranal; and a glycoside terpenoid, picrocrocin, responsible for saffron's coloring power, bitter taste, and aroma, respectively [3]. The quantities of these compounds in saffron are influenced by many factors, and the dehydration treatment necessary to convert* Crocus sativus* L. stigmas into saffron spice is one of the most important factors [4]. In order to optimize the dehydration process, saffron from India [5], Iran [6], Australia [7], Italy [8], Turkey [9], and Spain [10, 11], dried by shade drying, sun drying [5], solar drying [5], dehumidification drying [5], infrared drying [6], microwave drying [6], electric oven drying [5–7, 11], freeze drying [7, 9], hot air drying [10, 11], vacuum oven drying [5], or cross-flow oven drying [5], was studied. All the saffron samples dried by different methods in the reported papers were evaluated in terms of their quality determined by the ISO/TS 3632 or the quantities of the crocins, picrocrocin, and safranal determined by HPLC or GC.

**Figure 1:**
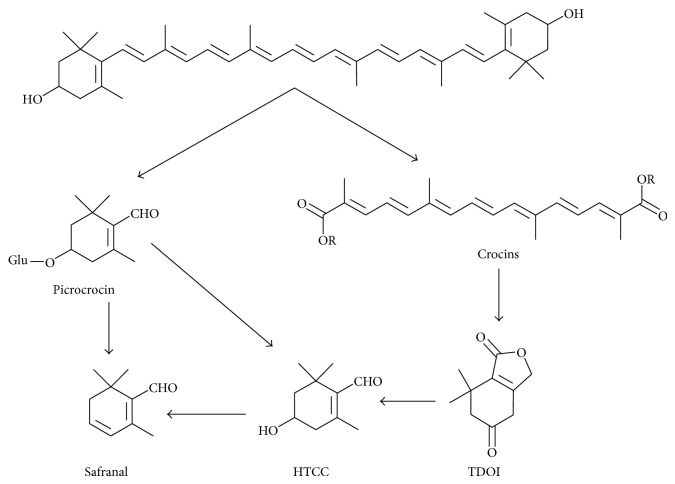
The possible pathways for the generation and degradation of the main compounds in saffron.

In addition, the possible pathways for generation and degradation of the main compounds in saffron were also studied to explain the change in quantity of these compounds during drying process. As shown in Figure 1 [4], the main compounds are formed from zeaxanthin which is broken down by an enzyme called CsZCD at both ends to generate crocetin dialdehyde and picrocrocin. Subsequently, picrocrocin is transformed by thermal treatment or alkaline-acid hydrolysis into safranal with HTCC as the potential intermediate compound for the synthesis of safranal from picrocrocin [4]. On the other hand, crocetin dialdehyde is oxidized and esterified by different glucosyl transferases to generate the crocetin esters which could also be precursor of safranal, converted into safranal by an enzymatic action or drying at high temperature as suggested by Carmona et al. [4]. In this possible pathway, TDOI is an important intermediate compound.

In China, saffron is a well-known traditional Chinese medicine to stimulate blood flow and relieve pain by removing stagnated blood. It has been successfully cultivated in China since the 1970s. In 2013, the total production of saffron was about 1 ton, and about 90 percent of Chinese saffron cultivated was in Jiande City in the Zhejiang Province of China. More and more saffron has been cultivated in China because of the increasing saffron demand.

The drying process of Chinese saffron is still an important part of research. According to the statutory standards of Zhejiang, saffron used for TCM should be dehydrated at low temperatures (<60°C). However, there are different drying processes including microwave drying, vacuum oven drying, and electric oven drying where the drying temperature is higher than 60°C. These three drying methods have been discussed in reported papers. Fresh saffron from India was dried by the vacuum oven drying method, and the crocins content was determined by UV at 440 nm by ISO/TS 3632 in 1996. In 2012, fresh saffron from Iran was dried by microwave, and the levels of crocins, picrocrocin, and saffron were determined by ISO/TS 3632. However, the quantities of picrocrocin and saffron determined by UV at 254 nm and 330 nm are not accurate because of the interference of cis-crocins and flavonoids in saffron; therefore, the research methods used for analysis need to be improved. In this paper, the HPLC fingerprints of Chinese saffron samples dried by different methods will be established, and the relation between the drying process and compounds' quantities will be discussed. In addition, correlations between the levels of the main constituents and antioxidative function will be studied in order to find out more about the antioxidant compounds in saffron.

## 2. Materials and Methods

### 2.1. Material Collection

All fresh saffron for dehydration research was harvested in the Sandu Town of Jiande City in the Zhejiang Province in China on October 15, 2013. Stigmas for the experiments were separated by hand indoors on the same day from a random selection of the picked flowers. The samples were then dehydrated by different methods immediately. It should be noted, however, that no attempt was made to test for the effect of harvest time in this study and that a variety of factors such as weather condition and the exact time between flower picking and stigma removal would vary between harvest dates.

### 2.2. Saffron Dehydration Process

The fresh samples were dried by different methods including electric oven drying (S1–S6), vacuum oven drying at 0.1 Mpa (Z1–Z6), or microwave drying (W1–W4). Details of the dehydration processes of all samples are shown in Table 1. All experiments were repeated three times, and the samples were kept in a cool and dry place at room temperature prior to chemical analysis.

### 2.3. Sample Extraction

Saffron was ground with an agate pestle and mortar and passed through a 0.4 mm sieve. Then ten milligrams of saffron was extracted by ultrasonic assist for 90 min in 10 mL of methanol-water (1 : 1) and filtered through a PVDF filter of 0.45 *μ*m. The whole process was carried out in darkness.

### 2.4. Analytical Determinations

#### 2.4.1. RP-HPLC-DAD Analysis

The HPLC fingerprint of saffron was carried out with the Agilent 1260 HPLC, consisting of four pumps, an autosampler, and a diode array detector. Separation was carried out on an Agilent C_18_ (250 mm × 4.6 mm i.d., 4 *μ*m) column. Twenty microliters of the extract was injected into the chromatograph at 30°C. The solvents were methanol (A), acetonitrile (B), and water (acidified with formic acid, 0.2%) (C). The following gradient was used: 10% A, 13.5% B, and 76.5% C to 100% A, 0% B, and 0% C in 60 min. The flow rate was 1 mL/min. The DAD detector was set at a full spectrum (200–600 nm) and at 440, 254, and 312 nm for crocins, picrocrocin, and safranal, respectively.

The crocetin esters quantification was carried out by taking into account the molecular coefficient absorbance of trans-crocins (89000 at 440 nm) and cis-crocins (63350 at 440 nm) and expressed as the percentage of each crocin in relation to the total crocins content. Quantitative determinations of picrocrocin, HTCC, and safranal were made as the ratios of each compound integration area obtained at the wavelength of maximum absorbance of the respective compound to the corresponding saffron concentration (mg/mL) (*A*_comp_/*C*_saffron_). Data reported represents the average of three sample replicates.

#### 2.4.2. HPLC-MS

Qualitative on-line HPLC–ESI-MS analyses were performed using an Agilent 1100 HPLC coupled to a Thermo Electron LCQ Deca IT spectrometer. The chromatographic conditions were as described above for HPLC-UV analyses at 440 nm. The mass spectrometer was operated in the positive and negative ion mode under the following conditions: capillary voltage, ±3 kV; capillary temperature, 195°C; nebulizer pressure, 30 psi; the auxiliary and sheath gas (nitrogen) flow rate, 12 L/min; and drying gas temperature, 350°C. Data were acquired in the MS scanning mode with scan ranges of 100–1500 *m*/*z*.

#### 2.4.3. UV-Vis Spectrophotometry

The UV-Vis spectra of all of the extracts after proper dilution were recorded in the region 200–600 nm with a spectrophotometer (Shimadzu UV 2450, Kyoto, Japan) equipped with quartz cells (1 cm × 1 cm × 4 cm). Absorption measurements in triplicate were obtained for each solution and ranged from 0.1 to 1.5 (photometric range of the instrument: −0.5–3.99). The results were expressed according to ISO/TS 3632(2003).

#### 2.4.4. DPPH Free Radical-Scavenging Assay

The DPPH free radical-scavenging activities of saffron extracts were determined according to the method described by Leong and Shui [12] with modifications. Briefly, a 2 mL solution of the samples at different concentrations was mixed with 2 mL of 0.06 mM DPPH solution. The absorbance at 517 nm was measured after keeping the mixtures in the dark for 30 min at room temperature. The percentages of DPPH reduced (AA) were calculated according to the following equation:

(1) $$\begin{array}{c} \text{AA}=100*\frac{\left[A_{0}-\left(A_{1}-A_{s}\right)\right]}{A_{0}}, \end{array}$$

where *A*_0_ is the absorbance of the control solution containing only DPPH, *A*_1_ is the absorbance of the DPPH solution containing samples, and *A*_*s*_ is the absorbance of the sample solution without DPPH. The antioxidant activity of the samples, expressed as IC_50_ (mg/mL), was compared with standard antioxidants such as ascorbic acid. The experiment was carried out in triplicate, and the results are mean values.

## 3. Results and Discussion

### 3.1. Saffron Quality Characteristics

According to the rules of ISO/TS 3632-2(2003), saffron samples were initially analyzed [13]. The moisture and volatile matter contents of all samples were less than 12%. Classification of the samples examined in this study was based on the limits set by ISO/TS 3632 regulations. All samples fell into the first ISO category (see Table 1).

### 3.2. HPLC Fingerprint of Saffron from China

**Figure 2:**
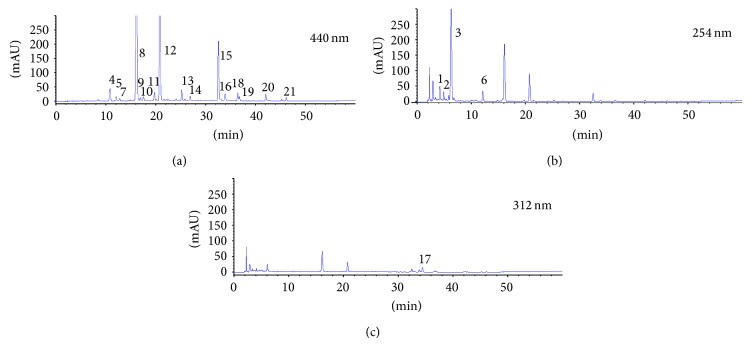
The fingerprints of saffron.

The HPLC fingerprints of saffron from different locations in China have been established by Wang et al. [14], but only 14 detectable peaks were obtained. In order to get more chemical information about saffron, a modification of the HPLC fingerprint determination was established by optimizing the extraction process and chromatographic conditions. We selected 440 nm, 312 nm, and 254 nm as the detector wavelengths, and 21 detectable peaks were obtained. The number of detected peaks and the peak resolution values obtained by this chromatographic method were better than those previously obtained by Wang et al. [14] (see Figure 2).

### 3.3. Qualitative Identification of Chemical Components in Saffron

Several papers have been published to qualitatively identify the chemical components in saffron. In this paper, six different components belonging to the crocetin esters family were identified at 440 nm by comparing their UV and MS data to data previously described in the literature [15]. Picrocrocin, HTCC, and safranal were identified by comparison of their retention time and UV data to data previously described in the literature. The peak identification is shown in Table 2, and nomenclature for the crocetin esters was adopted from Carmona et al. [10]. According to our analysis, different saffron samples did not differ in their chemical composition but did differ in the concentration of each component [15].

### 3.4. Influence of Dehydration Methods and Process on Chinese Saffron

#### 3.4.1. Dehydration Efficiency

In this paper, the fresh samples were dried by three different methods, including electric oven drying, vacuum oven drying, and microwave drying. In the microwave drying method, the dehydration efficiency is related to microwave power. It only takes 3 min at 600 W to dry the saffron when the moisture is less than 12% and 6 min at 400 W. The dehydration efficiency of vacuum oven drying is higher than that of electric oven drying because of vacuum pressure. It only takes 30 min at 50°C in vacuum oven drying to dry the saffron when the moisture is less than 12% while the electric oven drying takes 45 min.

#### 3.4.2. Electric Oven Drying Method

**Figure 3:**
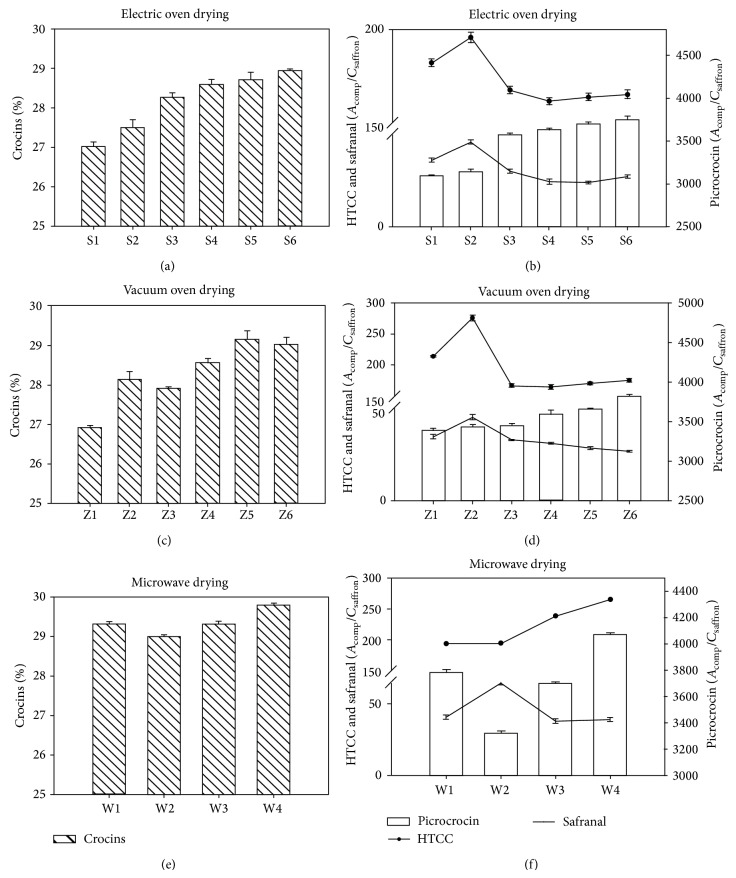
The crocin, safranal, and picrocrocin quantities in saffron dried by different methods.

As shown in Figures 3(a) and 3(b), the crocin content increased by 1.81% when the drying time was changed from 50 min to 65 min at 50°C; the peak areas of picrocrocin, HTCC, and safranal increased by 1.34%, 7.48%, and 26.90%. When the drying time was changed from 45 min to 60 min at 60°C, the crocin content increased by 1.13% and the peak area of picrocrocin increased by 1.37% while the HTCC and safranal decreased by 3.65% and 23.13%. The crocin content increased by 0.77% when the drying time was changed from 30 min to 45 min at 70°C; the peak areas of picrocrocin, HTCC, and safranal increased by 1.29%, 0.86%, and 13.18%.

#### 3.4.3. Vacuum Oven Drying Method

As shown in Figures 3(c) and 3(d), the crocin content increased by 4.49% when the drying time was changed from 30 min to 45 min at 50°C, and the peak areas of picrocrocin, HTCC, and safranal increased by 1.13%, 28.80%, and 30.90%. When the drying time was changed from 30 min to 45 min at 60°C, the crocin content increased by 2.37%, and the peak area of picrocrocin increased by 4.05%, while the HTCC and safranal decreased by 1.61% and 5.70%. The crocin content decreased by 0.41% when the drying time was changed from 30 min to 45 min at 70°C, and the peak areas of picrocrocin and HTCC increased by 4.62% and 2.78%, while the safranal decreased by 6.10%.

#### 3.4.4. Microwave Drying Method

As shown in Figures 3(e) and 3(f), the crocin content increased by 1.60% when the drying time was changed from 6 min to 10 min at 450 W, and the peak areas of picrocrocin, HTCC, and safranal increased by 10.14%, 11.16%, and 3.11%. When the drying time was changed from 3 min to 6 min at 600 W, the crocin content decreased by 1.10%, and the peak area of picrocrocin increased by 13.84%, while the HTCC and safranal decreased by 0.55% and 58.59%.

#### 3.4.5. Change Mechanism

According to the research of Gregory et al. [7], Carmona et al. [10], and Tsimidou and Biliaderis [16], the crocins and picrocrocin are formed by the oxidative degradation of zeaxanthin (as shown in Figure 1) in low temperature. When the high temperature was employed in drying process, the crocins and picrocrocin in chromoplast would be released because of the destruction of chromoplast. The crocins and picrocrocin were also the thermolabile substances, and they were converted to safranal by enzymatic or dehydration process and HTCC was one of the important compounds in the process. In a word, the generation and degradation of crocins, picrocrocin, and HTCC were conducted at the same time, but the generation and degradation rate was different which was related to temperature. With this in mind, it is easy to explain the variations of these compounds in the drying process.

### 3.5. Correlations between Content of Main Constituents and Antioxidative Function

The scavenging effects on DPPH radicals of the different saffron samples under investigation are shown in Table 1. The multivariant correlation analysis between antioxidant activity and As/C samples of 21 common characteristic peaks in the HPLC fingerprints was achieved by SPSS statistics software (Table 2). The results showed that peaks 8, 12, and 20 in HPLC fingerprints possess a close correlation with the antioxidant activity of saffron, and these peaks might correspond to the main antioxidant components. These results are similar to the work of Chen et al. [1], which showed that the antioxidant of the crocins-1 is almost equal to that of crocins-2 and is much stronger than that of crocins-3.

Curiously, the antioxidant value of Z3 was smaller than those of S1, S2, Z1, and Z2 (Table 1), while the content of most of the peaks in S01, including crocins-1 and crocins-2, was larger than those in S1, S2, Z1, and Z2 (Table 1). Some possible explanations are listed as follows: (1) S1, S2, Z1, and Z2 might possess other strong antioxidant components which cannot be monitored at 440, 254, and 312 nm; (2) the antioxidant effect of saffron is based on the synergic effect of mass constituents [17].

## 4. Conclusion

The drying process had a great influence on the chemical content and antioxidant activity of saffron. The chemical contents in the samples treated by microwave drying were higher than the other two methods and the time spent in drying process was also less, but the antioxidant activity of these samples was not stronger, which means that other chemical compounds were formed in the samples treated by electric oven drying and vacuum oven drying processes. This work clearly showed that the saffron quality which is influenced by drying methods should be evaluated by chemical contents and pharmacological activities.

## Figures and Tables

**Table 1:** The contents of crocins, safranal, and picrocrocin in saffron determined according to ISO/TS 3632 (2003).

Number	Drying treatment type	Drying temperature and duration	E440 nm	E330 nm	E254 nm	Quantity of crocins-1	Quantity of crocins-2	IC_50_
S1	Electric oven drying	50°C for 50 min	210.42 ± 0.9	26.75 ± 0.41	73.37 ± 0.36	16.84 ± 0.05	6.82 ± 0.05	19.38 ± 0.71
S2	Electric oven drying	50°C for 65 min	214.20 ± 1.47	28.49 ± 0.35	81.14 ± 0.24	17.36 ± 0.15	6.78 ± 0.04	20.01 ± 0.68
S3	Electric oven drying	60°C for 45 min	219.95 ± 0.88	30.29 ± 0.27	81.16 ± 0.23	17.91 ± 0.10	7.36 ± 0.05	23.70 ± 0.67
S4	Electric oven drying	60°C for 60 min	222.62 ± 1.01	29.87 ± 0.13	83.04 ± 0.28	17.57 ± 0.02	7.80 ± 0.11	18.35 ± 0.82
S5	Electric oven drying	70°C for 30 min	223.36 ± 1.46	33.68 ± 0.04	85.23 ± 0.21	17.84 ± 0.06	7.25 ± 0.09	18.02 ± 0.78
S6	Electric oven drying	70°C for 45 min	224.72 ± 0.36	32.75 ± 0.37	87.01 ± 0.31	17.73 ± 0.11	7.68 ± 0.07	19.53 ± 0.78
W-1	Microwave drying at 600 W	3 min	228.31 ± 0.46	31.72 ± 0.21	87.70 ± 0.62	18.42 ± 0.09	7.73 ± 0.02	23.24 ± 0.82
W-2	Microwave drying at 600 W	6 min	225.39 ± 0.36	35.62 ± 0.24	91.25 ± 0.14	18.23 ± 0.07	7.17 ± 0.03	22.81 ± 0.76
W-3	Microwave drying at 450 W	6 min	231.97 ± 0.22	31.93 ± 0.32	87.07 ± 0.42	18.34 ± 0.04	7.72 ± 0.08	30.46 ± 0.92
W-4	Microwave drying at 450 W	10 min	228.29 ± 0.47	32.85 ± 0.42	89.68 ± 0.21	18.69 ± 0.08	7.94 ± 0.06	27.02 ± 0.77
Z-1	Vacuum oven drying at 0.1 Mpa	50°C for 30 min	209.54 ± 0.34	29.98 ± 0.16	87.10 ± 0.07	16.67 ± 0.06	7.23 ± 0.07	22.65 ± 0.75
Z-2	Vacuum oven drying at 0.1 Mpa	50°C for 45 min	218.58 ± 1.57	37.35 ± 0.05	91.09 ± 0.29	16.86 ± 0.20	7.30 ± 0.05	23.63 ± 0.98
Z-3	Vacuum oven drying at 0.1 Mpa	60°C for 30 min	217.21 ± 0.31	31.25 ± 0.19	86.19 ± 0.21	17.56 ± 0.01	7.31 ± 0.01	14.46 ± 0.74
Z-4	Vacuum oven drying at 0.1 Mpa	60°C for 45 min	222.13 ± 0.78	27.53 ± 0.28	81.66 ± 0.06	17.47 ± 0.08	7.73 ± 0.01	26.52 ± 0.95
Z-5	Vacuum oven drying at 0.1 Mpa	70°C for 30 min	226.89 ± 1.67	29.09 ± 0.75	83.90 ± 0.33	18.19 ± 0.19	7.55 ± 0.03	30.41 ± 0.82
Z-6	Vacuum oven drying at 0.1 Mpa	70°C for 45 min	225.95 ± 1.4	28.94 ± 0.13	82.30 ± 0.51	18.15 ± 0.12	7.76 ± 0.06	28.67 ± 0.90

**Table 2:** The relation of peaks to antioxidant value.

Number of peaks	Constituent	Correlation coefficient	Number of peaks	Constituent	Correlation coefficient
1	/	0.196	12	Trans-3-Gg	0.530
2	/	0.401	13	Trans-2-gg	0.273
3	Picrocrocin	0.432	14	/	0.184
4	Trans-5-tG	0.399	15	Trans-2-G	0.190
5	HTCC	0.374	16	Cis-4-GG	0.079
6	/	0.311	17	Safranal	0.033
7	/	0.101	18	Cis-3-Gg	0.329
8	Trans-4-GG	0.587	19	/	0.390
9	/	0.311	20	/	0.513
10	/	0.469	21	/	0.313
11	Trans-4-ng	0.416			
